# Infants' Daily Experience With Pets and Their Scanning of Animal Faces

**DOI:** 10.3389/fvets.2018.00152

**Published:** 2018-07-10

**Authors:** Karinna Hurley, Lisa M. Oakes

**Affiliations:** ^1^Center for Mind and Brain, University of California, Davis, Davis, CA, United States; ^2^Human Development, University of California, Davis, Davis, CA, United States; ^3^Department of Psychology, University of California, Davis, Davis, CA, United States

**Keywords:** infant development, pets, experience, cognitive development, human-animal interaction, face processing

## Abstract

Very little is known about the effect of pet experience on cognitive development in infancy. In Experiment 1, we document in a large sample (*N* = 1270) that 63% of families with infants under 12 months have at least one household pet. The potential effect on development is significant as the first postnatal year is a critically important time for changes in the brain and cognition. Because research has revealed how experience shapes early development, it is likely that the presence of a companion dog or cat in the home influences infants' development. In Experiment 2, we assess differences between infants who do and do not have pets (*N* = 171) in one aspect of cognitive development: their processing of animal faces. We examined visual exploration of images of dog, cat, monkey, and sheep faces by 4-, 6-, and 10-month-old infants. Although at the youngest ages infants with and without pets exhibited the same patterns of visual inspection of these animals faces, by 10 months infants with pets spent proportionately more time looking at the region of faces that contained the eyes than did infants without pets. Thus, exposure to pets contributes to how infants look at and learn about animal faces.

## Introduction

Many families with children have pets (1–3), and there has been significant interest in the connection between experience with animals and development in childhood (4–8). However, few studies have considered the impact of exposure to pets on very young infants (9). Instead, the vast majority of work on how exposure to animals influences development has focused on older children and, often, in therapeutic settings (4, 10, 11). The lack of work on the period of infancy is surprising because it is a developmental period profoundly influenced by experience. For example, experience with particular sounds, faces, and objects contribute to infants' rapidly developing abilities in language (12), facial perception (13), and categorization (14). Why has the effect of pets on infants' development been so neglected? One possibility is that because households without children often have high levels of pet ownership (15, 16) people assume that most families with infants are unlikely to have pets, and thus there are few opportunities for infant development to be shaped by pets. Another possibility is that research on the effect of pet experience on development has not focused on typical cognitive development, as the examples given for the effect of languages, faces, and categorization.

Here we address both of these possibilities. First, we present data on the prevalence of pets in homes with infants between 4 and 12 months of age. These data provide an important context for why researchers should focus on the influence of pets on development during this age range. To preview our findings, we observe that families with infants have companion dogs and cats at similar rates as have been reported for families with older children (17). Thus, there is no reason to assume that infants have less exposure to pets than do children at other developmental stages.

Next, we examine the effect of pet exposure on one aspect of typical cognitive development in infancy, their learning of *animal faces*. Thus, our work will fit in the context of findings that infants' developing face processing is related to their experience with faces of a particular gender, race, or species. For example, infants have a processing advantage for female faces (18–20), perhaps because most infants have female primary caregivers (21), and therefore, in general, have more experience with female faces. By 3 months infants show preferences for own-race faces over those from unfamiliar races (22–24), presumably reflecting, at least in part, their daily experience with faces of a particular (parents') race. In addition, experience shapes the *development* of infants' face processing. Although 3-month-old infants discriminate between individual faces from both their own (parents') racial group as well as from other less familiar racial groups, 9-month-old infants discriminate faces only from their own racial group (25). Similarly, whereas 6-month-old infants discriminate both individual human and monkey faces, 9-month-old infants discriminate only individual *human* faces and are unable to discriminate between individual monkey faces (26).

We extend this work to examine the effect of daily exposure to companion dogs and cats on infants' developing processing of animal faces. Providing infants with daily experience with monkey faces between 6 and 9 months helped them maintain the ability to discriminate monkey faces (27, 28), and this effect is particularly robust when that daily experience with each animal emphasized animals as an individuals [i.e., looking at pictures of named individuals (28)]. Exposure to a pet in the home, which emphasizes that pet as an individual (i.e., pets are named, they are talked to), may influence infants' perceptual processing of face stimuli similar to that pet. Thus, our results will allow us to generalize the effect of this artificial experimental manipulation to a naturalistic difference that occurs in infants' daily life. Family pets have the potential to have a profound effect on infants' development. Not only do infants with and without pets differ in their amount of exposure to animals, their experience with pets likely differs in other ways given the interactive social nature of domestic animals (29–33) and the fact that pets commonly are considered family members (34–38).

The work presented here builds on previous findings demonstrating that infants who live with indoor pets perceive and learn about images of dogs and cats in the lab differently than infants who do not live with indoor pets (39–43). For example, Kovack-Lesh et al. (41, 43) found that found that 4-month-old infants with pets responded differently in a categorization task than did infants without pets, at least if they engaged in high levels of looking back-and-forth between the two images during familiarization. Thus, observed differences in infants' responding during test trials appears to have been a function of their past experience. Other work points to differences in how infants actually *approach* stimuli as a function of their pet experience. Hurley et al. (39) observed that 6-month-old infants with pet experience engaged in more looking and comparison when viewing images of animals than did infants without pet experience, consistent with other findings that infants are more interested in stimuli relevant to their past experience (19, 44). Examinations of eye-movements of 4-month-old infants as they inspected individual images of cats and dogs revealed that infants with pets looked more at the informationally-rich head regions than did infants without pets (40, 42). Thus, experience with dogs and cats in the home appears to have translated into differences in attentional biases when infants processed images similar to that experience. Hurley and Oakes (40) further showed that infants with and without pets did not differ in their visual inspection of human faces and vehicles, suggesting that the effect of such animal experience was specific to images of animals that were similar to the animals common in the everyday experience of infants with pets.

The current work addressed several important unanswered questions. First, none of the previous studies of pet experience examined *age-related changes* in the effect of pet experience on infants' visual processing of animals. All of the existing work in this area has examined the relation between pet experience and visual processing of animal images in infants at a single age (39–43). We predict from the work on infants' processing of human faces, however, that pet experience will differentially influence how younger and older infants visually process images of animals, presumably as both the result of older infants having more experience—and that experience having more time to influence processing—than younger infants and the result of the effect of experience on development at early ages having a cascading effect on later developing skills and abilities. As described in more detail in the General Discussion section, we assume that daily experience with a pet helps to shape the attentional strategies infants adopt when looking at animal images. Thus, we anticipate that there will be differences in how older infants visually explore or scan images of animal faces as a function of pet experience, although there may be few, if any, differences in how younger infants visually explore or scan animal faces. We tested this prediction by observing separate groups of 4-, 6-, and 10-month-old infants' looking at animal faces. These are key ages in the work on changes in infants' processing of human faces.

A second question we addressed in this investigation is whether the effect of experience would be observed for infants' processing of animal *faces*. All the previous investigations of pet experience on infants' processing of dog and cat images have used representations of whole animals as stimuli. Although this work has shown that infants with pets have a stronger bias to look at the head and face region of these images (40, 42), we do not know whether differences will be observed for how infants process the *faces* of animals. Previous work suggests head regions are especially informative for infants' processing of animal images (45, 46), and Mareschal et al. (47) established that infants are sensitive to variations in the facial features of cats and dogs. Moreover, if the effects of experience on infants' processing of human faces reflect general processes, then we should see similar effects for the effect of experience on infants' processing of non-human faces. For these reasons, in the present investigation we presented infants with images of novel animal faces.

Finally, we asked how infants' pet experience extended to their processing of different types of animals. The previous work has focused on infants' visual cognitions of images of dogs and cats (39–43). Although (40) showed that the effect of pet experience did not extend to images of human faces and vehicles, we do not know how infants' pet experience influences their processing of other kinds of animals. Therefore, we presented infants with images of dogs and cats, that are likely highly familiar to infants (particularly infants who have pets at home) and images of animals monkeys and sheep, that are likely relatively unfamiliar to infants.

## Experiment 1

The goal of Experiment 1 was to document the prevalence of pet ownership in families with infants between 4 and 12 months of age. This Experiment will demonstrate that many infants are exposed to companion dogs and cats in their daily lives, and that there are significant opportunities for pets in the home to shape development in infancy.

Both Experiments 1 and 2 were carried out in accordance with the recommendations of the Institutional Review Board of the University of California, Davis. The protocols were approved by the Institutional Review Board of the University of California, Davis. All subjects gave written informed consent in accordance with the Declaration of Helsinki.

### Methods

#### Participants

Between 12/13/2007 and 12/12/2014, we asked the parents of 1,270 infants between 106 and 320 days of age (*M* = 176.70, *SD* = 61.13) who were visiting our lab about the companion dog and cat animals who live in their homes. There were 648 boys and 622 girls. Infants were full healthy, typically developing full-term infants recruited from the greater Sacramento Valley region of Central California.

Names of potential participants were initially obtained from the State Vital Records office. All parents who lived within a ~30-min drive from the lab were sent informational packets describing our work and a general invitation to participate in studies, and parents who were interested in volunteering contacted us. Infants were recruited for this investigation solely based on age, and any infant in our pool who was born full term and who was healthy and typically developing was recruited to participate in this study via phone call or e-mail (depending on parental stated preference when they volunteered). Parents and infants received a certificate and a t-shirt, toy, or book as a thank-you for participation.

The parents of our sample were highly educated. Of the 1,256 mothers who reported their education, all but 17 completed high school, all but 70 had at least some college, and 847 had earned at least a bachelor's degree. Of the 1,196 parents who reported their infants' race, 852 reported their infant to be White, 36 reported their infant to be Black, 56 reported their infant to be Asian, 232 reported their infants to be of mixed race, and 20 reported their infants to be Native Hawaiian, American Indian, or other. Of the 1,196 parents who reported it, 323 indicated that their infant was Hispanic (165 White, 69 mixed race, 66 with no race reported, and the remaining were Black, Asian, Native Hawaiian, American Indian, or other race). Thus, our infants represented the diversity of the community.

#### Procedure

When infants came to our lab to participate in a study of infant cognition, parents completed a questionnaire about family demographics (see Appendix). In this questionnaire, parents reported infant birthdate, due date, sex, race, and mother's education and the age of any older siblings. In addition, they reported on their infants' pet experiences by replying verbally to the following question: “Do you have pets?” If the answer was yes they were asked about the number and type as well as whether the pet(s) lived indoors with the family.

### Results and discussion

To examine the likelihood of proportions, we conducted binomial probabilities of observing the number of occurrences or more given the sample size. We compared the difference in proportions between two groups (e.g., infants with and without siblings, Hispanic vs. non-Hispanic families) with z-ratios for the difference between two independent proportions. We used two-tailed tests to evaluate these z-ratios. All binomial and z-tests were conducted using vassarstats.net. We compared group means on continuous variables (e.g., age, maternal education) using two-tailed *t*-tests independent groups, performed using IBM SPSS Statistics for Mac (Armonk, NY: IBM Corp). Our critical *p*-value for significance was 0.05, except as noted to correct for multiple comparisons.

Of the 1,270 parents who completed our questionnaire, 804 (63.31%) reported having a pet dog or pet cat (or both), a proportion that was significantly different from chance, binomial probability (804 or more out of 1270), *p* < 0.001 (see Table 1). Of the 1,253 families who reported whether or not their pet lived indoors, 696 (55.55% of the sample) reported having an indoor pet, a proportion that was significantly different from chance, binomial probability (696 or more out of 1253), *p* < 0.001. The numbers of families who had dogs and cats or both are presented in Table 1. Clearly, in our sample more families had dogs than cats; 387 62%) of the 626 families who had only dogs or cats had only dogs, a proportion that is significantly different from chance, *p* < 0.001. Thus, most of the families in our sample had one or more pet, and more than half of the infants in our sample had exposure daily to a pet in the home.

**Table 1:** Demographics of infants with and without pets in Experiment 1.

**Group**	***N***	**Number of boys**	**Average age in days (*SD*)**	**Infants with older siblings (of 1240 who reported sibling information)**	**Number years maternal education (*SD*)**
Pet	804	417 (52%)	176.08 (59.64)	381 (48%)	15.99 (2.05)
Cat only	239	120 (50%)	176.19 (61.23)	(105) 47%	16.46 (2.04)
Dog only	387	214 (55%)	174.23 (58.66)	(263) 48%	15.74 (2.07)
Dog and cat	178	83 (47%)	180.00 (59.75)	(75) 42%	15.87 (1.94)
No Pet	466	231 (50%)	177.76 (63.68)	250 55%	15.83 (2.16)
*Total*	1,270	648 (51%)	176.70 (61.13)	631 (51%)	15.93 (2.09)

To gain a clearer understanding of the frequency and type of pet ownership in this group, we provide in Table 1 demographics for families who had any dog or cat (indoor or outdoor) and families without any pets. As is clear from this table, the two groups of infants looked very similar; they both had approximately equal proportion of boys and girls and the average age of the samples did not differ. For the infants who had information about siblings reported, the proportion of infants without pets who had siblings was significantly greater than the proportion of infants with pets who had siblings, *z* = 2.23, *p* = 0.03.

Overall, maternal education did not differ for families who had pets compared to families who did not have pets, *t*_(1254)_ = 1.25, *p* = 0.21, *d* = 0.07. Mother's education was higher for families who only had cats than families who had no pets, *t*_(701)_ = 3.71, *p* < 0.001, *d* = 0.30, families who had only dogs, *t*_(617)_ = 4.23, *p* < 0.001, *d* = 0.35, and families who had both dogs and cats, *t*_(410)_ = 2.94, *p* = 0.003, *d* = 0.29. Mother's education did not differ between families without pets and families who had only dogs, *t*_(842)_ = 0.63, *p* = 0.53, *d* = 0.04, or who had both dogs and cats, *t*_(635)_ = 0.21, *p* = 0.84, *d* = 0.02. Similarly, maternal education did not differ between families who had only dogs and families who had both dogs and cats, *t*_(551)_ = 0.70, *p* = 0.48, *d* = 0.06. We also evaluated these differences for families who reported having indoor pets, and the patterns were identical.

Next we examined how pet ownership varied according to infant race, which is a proxy for the family race (in our sample, all infants have the same race as their parents; if the parents are of different races, the infant is reported as mixed race). For the present purposes we divided the infants into three groups according to reported race: White and not Hispanic, infants who were rated as neither White nor Hispanic, and infants who were reported as Hispanic regardless of race. The proportion of families in each of these groups that had pets is presented in Table 2.

**Table 2:** Race information for infants with and without pets in Experiment 1.

	**White/not Hispanic**	**Not White/not Hispanic**	**Hispanic**
Pet	474 (70%)	126 (50%)	195 (60%)
Cat only	155 (43%)	41 (33%)	42 (22%)
Dog only	203 (33%)	61 (48%)	116 (59%)
Cat and dog	116 (24%)	24 (19%)	37 (20%)
No pet	205 (30%)	127 (50%)	128 (40%)
Total	679	253	323

In our sample, the proportion of White/non-Hispanic families with pets was greater than the proportion of non-White/non-Hispanic families with pets, z = 5.67, *p* < 0.001, and than Hispanic families, z = 2.96, *p* = 0.003. More Hispanic families had pets than did non-White/non-Hispanic families, z = 2.53, *p* = 0.01. This is not due to the fact that most Hispanic families were White; 165 (51%) of the infants who were reported to be Hispanic were also reported to be White. In addition 95 (58%) of the White/Hispanic families had pets and 100 (63%) of the non-White/Hispanic families had pets. Thus, the differences appear to be a lower rate of pet ownership by families who are non-White and non-Hispanic. However, this finding would need to replicated in a larger, more representative sample before strong conclusions could be drawn about racial differences in pet ownership by families with infants.

What is clear from these data is that many infants have opportunities to learn from household pets, and that this is a naturally occurring difference in experience that could yield different developmental outcomes. Interestingly, infants were not more likely to have a pet *and* a sibling; more families in our sample with pets had only one child. In addition, although there were no overall differences in maternal education and the presence of a pet, maternal education was highest for families who had only cats than for any other group. These data are the first to our knowledge to describe aspects of the home context of infants under 1 year who do and do not live with pets.

## Experiment 2

Experiment 1 revealed that ~63% of the infants between the ages of 4 and 12 months living in our region have daily experience with pets. In Experiment 2, we asked how infants with this experience differed from infants without such experience in their processing of animal faces. Importantly, we examined the effect of animal experience across age, allowing us to determine whether infants with and without pets differed at all points in development, or whether the effect of such experience changed across this developmental period.

### Methods

#### Participants

The final sample included a total of 171 healthy, full-term infants with no known vision problem: 52 infants were 4 months old (*M* = 125.02 days, *SD* = 7.46 days; 24 girls and 28 boys), 57 infants were 6 months old (*M* = 184.91 days, *SD* = 7.92 days; 27 girls and 30 boys), and 62 infants were 10 months old (*M* = 304.11 days, *SD* = 7.89 days; 26 girls and 36 boys). The same self-report questionnaire was used here as in Experiment 1, probing the presence of pets and whether they lived indoors. These parental reports revealed that in our sample 64 infants did not have an indoor pet, 35 had only a cat or cats that lived indoors with the family, 52 had only a dog or dogs that lived indoors with the family, and 20 had both a cat or cats and a dog or dogs that lived indoors with the family; thus the proportion of infants in our sample with pets (63%) was similar to that in Experiment 1. Infants were recruited as described in Experiment 1. We tested 28 additional infants, but excluded their data from the final analyses due to fussiness or inattention (*N* = 8), equipment or experimenter error (*N* = 8), ambiguous pet status (i.e., an infant who had a dog for several months and then did not) (*N* = 1), or failure to provide useable data on the minimum number of trials (*N* = 11, see Data Processing section below).

In the final sample of 171 infants, 116 infants were reported to be White. The remaining infants were reported to be Asian (*N* = 4), Black or African American (*N* = 2), mixed race (*N* = 38), or other (*N* = 2); 9 parents did not report the race of their infant. Thirty-seven infants were reported to be Hispanic; of these infants 17 infants were White, 11 infants were mixed race, and 9 infants did not have their race reported. The sample was highly educated; of the 167 mothers who reported their educational background, all but one mother had completed high school, 47 had completed at least some college, and 113 had earned at least a bachelor's degree. Thus, the sample was demographically similar to that in Experiment 1.

#### Stimuli

**Figure 1:**
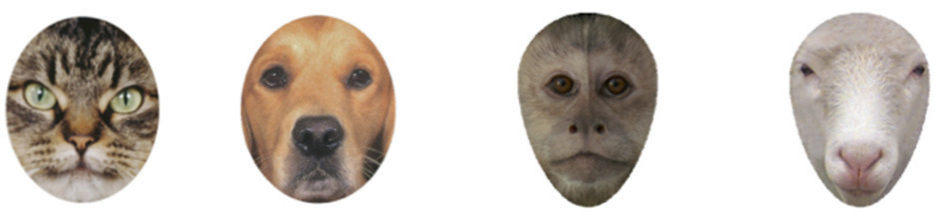
Examples of the four types of stimulus faces. A mask was imposed to reduce infants' attention to external features (such as ear shape).

Stimuli were digitized photographs of 12 different faces from each of four animal categories: cat, dog, monkey, and sheep (see Figure 1). Using these four types of images allowed us to compare infants' responding to both relatively familiar and relatively unfamiliar animal faces. Specifically, we selected cats and dogs because they are relatively familiar to infants (even infants who do not have a dog or cat as pet at home likely see one or both types of animals at the homes of friends and relatives, in the park, walking in their neighborhood, etc.), and we selected sheep and monkeys because they are relatively unfamiliar to infants. Thus, these four face types will allow us to determine how general any effect of pet experience is on infants' face scanning; if it extends only to familiar cats and dogs or even to unfamiliar sheep and monkeys. We selected monkeys and sheep specifically because they varied configurally, with the monkey faces being configurally more similar to cat faces (e.g., relatively large eyes, small noses) and sheep faces being configurally more similar to dog faces (e.g., smaller eyes at the top of the face, prominent snout with larger nose at the bottom of the face). Thus, this will allow us to determine if pet experience extends more to some configurations than to others. Finally, we selected sheep and monkeys faces because both species had been used in facial discrimination studies and thus good quality stimuli sets already existed.

Sheep faces came from a photograph stimuli set previously used to study facial discrimination in infants' and adults' (48) as well as in sheep (49). Monkey faces came from a photograph stimuli set used to understand facial discrimination in monkeys (50, 51). Cat and dog photographs were gathered from breed books and cropped to match in size in Adobe Photoshop. All faces were front-oriented, symmetrical, and similar in breed (dogs were either Golden Labradors or Golden Retrievers) species (all monkeys were tufted capuchins), or coloring and marking (all sheep where white and all cats were brown tabbies). Using Adobe Photoshop, an oval mask was overlaid on the images to make the external contours of the images identical within face type and similar across faces, similar to the mask used in Chien et al. (52). Thus, differences in infants' looking or scanning would not reflect differences in face shape, protrusion of ears, etc., but rather would primarily reflect differences in internal features, such as the prominence of the nose, the top-heaviness, etc. Due to differences in the overall shape of the different faces we created two masks; one mask for the dog and cat faces and another for the sheep and monkey faces. The mask covered the ears of all animals (see Figure 1). Images were ~38 cm × 25 cm in size, subtending ~21.5 by 14.25 degrees visual angle.

#### Apparatus

A Dell computer was used to present the stimuli and control the experiment. Stimulus images were presented side-by-side in the center of a 37-inch LCD TV monitor (19:9 aspect ratio), and subtended ~21.5 by 14.25° visual angle at a viewing distance of 100 cm. Eye gaze was recorded using an Applied Science Laboratory (ASL) pan/tilt R6 eye-tracker controlled by a second Dell computer. An eye-camera located at the bottom and center of the monitor focused on the infants' right eye; using the image from this camera, the eye-tracker calculated the location of infants' fixations from the reflections of an infrared light source of the cornea and pupil. A wide-angle camera was affixed to the eye-camera to provide an image of infants' heads and torsos. A sensor, attached to an infant-sized headband, was positioned above the right eye and was used to locate the infants' head in a magnetic field produced by a generator located directly behind the parents' chair. This position was communicated to the eye-tracker, which, if necessary, was used to adjust the camera to refocus the infants' eye (e.g., if the infant looked at the parent and then back at the screen). A white curtain separated the infants from the observers and equipment.

#### Procedure

Infants sat on their parents' lap in a dimly lit room ~100 cm from the monitor and ~75 cm from the eye-camera. Parents wore occluding glasses in order to reduce any bias their reaction to the stimuli could have on infants' looking. Sessions began with a five-point calibration protocol in which a looming circle was presented at each point: (1) 11.5° above and to the left of the central fixation point, (2) 11.5° above and to the right of the central fixation point, (3) at the central fixation point, (4) 11.5° below and to the left of the central fixation point, and (5) 11.5° below and to the right of the central fixation point. As infants fixated at each point an experimenter pressed a key on the computer to record the relative locations of the corneal and pupil reflections when the infant was fixating on that known location. This information was used to calculate the point-of-gaze (POG) for each data sample during the experiment.

Immediately after calibration the experimenter initiated the experimental paradigm. Each trial began with a geometric colored shape (e.g., a purple diamond, green triangle, yellow star) presented at the central fixation point; the shape continuously loomed for 800 ms (from 0° × 0° visual angle to ~16° × 16° visual angle) and was accompanied by a randomly selected sound (e.g., buzz, beep, ding). When infants fixated this stimulus (as indicated by cross-hairs superimposed on the stimulus by the ASL eye-tracking system) the experimenter pressed a computer key to initiate the start of an experimental trial.

The experimental trials were 5 s in duration, and on each trial a pair of images from the same category was presented (e.g., two dogs, two cats, two sheep, or two monkeys). We presented two images on each trial, center-to-center distance was 22° (the center of each image was ~11.5° to the left or right of midline). Each trial was initiated when infants looked at an attention getter at *center* of the monitor; thus when the stimuli were first presented, infants were fixating the center of the monitor and they had to move their eyes from fixation to look at either image. A bias to look at a particular region (e.g., eyes, nose) therefore could not reflect infants simply maintaining fixation in the location where the stimulus happened to be presented; rather any observed bias will reflect infants' selecting that region and maintaining their attention to it.

We created a custom program in Adobe Director to control stimulus presentation and randomly order image pairs in blocks of four trials. Each block contained one trial with a pair of dogs, one trial with a pair of cats, one trial with a pair of sheep, and one trial with a pair of monkeys. Thus, infants saw a pair of images from each animal category in each block of four trials. On each trial, a randomly selected clip of classical music (Bach, Beethoven Mozart, Pachelbel, Vivaldi, or Ravel) was played to aid in keeping infants' attention.

If the infant became uninterested in general and looked away from the monitor, the experimenter could present one of several stimuli to recapture their attention. These stimuli included sequences of randomly chosen clips of children's television shows (Teletubbies, Blues Clues, Sesame Street), a cartoon of animated animals singing, a series of pictures of babies accompanied by classical music, and the calibration stimuli. Key commands in the computer program were used to present the stimuli and allowed the experimenter to present any of the attention-getting stimuli between trials if infants' attention needed to be redirected to the center of the screen. There were a maximum of 264 experimental trials, and trials were presented until infants showed signs of disinterest in looking at screen (e.g., fussing, looking at the parent, refusing to look at the screen).

### Results

#### Data processing

**Figure 2:**
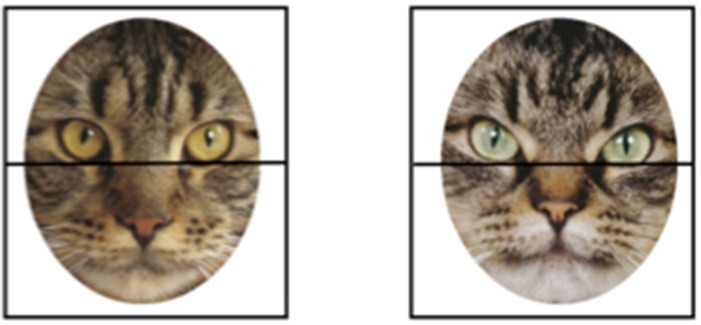
An example of one possible pair of stimuli presented on a single (cat) trial. To illustrate how we evaluated infants' looking times, Areas of Interest (AOIs) corresponding to the top and bottom halves of the faces are superimposed on the images.

Data processing was similar to that reported in Hurley et al. (40). The point of gaze data was recorded at a rate of 60 Hz, using an online average of 4 samples (the current sample and the 3 previous samples) to minimize noise in the data. In addition, a blink filter was implemented in which pupil loss of fewer than 12 samples was considered a blink. The horizontal and vertical position of the gaze was recorded at each sample with a code to indicate which type of stimulus was presented on each trial. Data were first processed using the software program ASL Results to parse the datastream into trials. Next, we used custom a Matlab routine to determine how may samples fell into prespecified Areas of Interest (AOIs). We evaluated infants' looking in four AOIs: the top and bottom halves of each of the two stimuli presented side-by-side (see Figure 2). This approach allowed us to have the same AOIs across faces and species, while having one AOI contain the eye region, known to be important for face processing (53). This approach—of dividing the face into upper and lower halves—has been used in other studies of face processing (54).

The number of samples in each AOI was converted to *duration* for analysis. We included in the analyses any trial in which at least 200 ms of looking was recorded; across all infants at all ages the analyses are based on an average of 28.49 trials per infant (Range = 4–105, *SD* = 14.85). All infants who contributed at least one trial of each type (cat, dog, monkey, and sheep) that met this criterion were included in the final analyses (as described in the Participants section, 11 infants failed to meet this criterion).

#### Analysis plan

We tested our hypotheses by examining differences in infants' preferences for the *top half* of the faces. To examine how infants' scanning of these faces varied by age and pet status we calculated infants' preference for the *top half* of each type of face. If infants focus more on the eye-region on our faces, as is typical when young infants scan human faces (53), we should see a strong preference for the top half of the faces. If infants scan more broadly—a pattern exhibited by older infants when exhibiting human faces (55)—will should see a weaker top-half preference. To evaluate any effect of age, pet experience, or type of face on infants' top half preference, we conducted Analyses of Variance (ANOVAs) comparing the top half preferences. We conducted follow up comparisons for any significant effects using *t*-tests, adjusting our criterion of significance to control for multiple comparisons. We also examined infants' preference for the top half by comparing their preferences using one-sample *t*-tests and Bayes Factors.

#### Analyses

**Figure 3:**
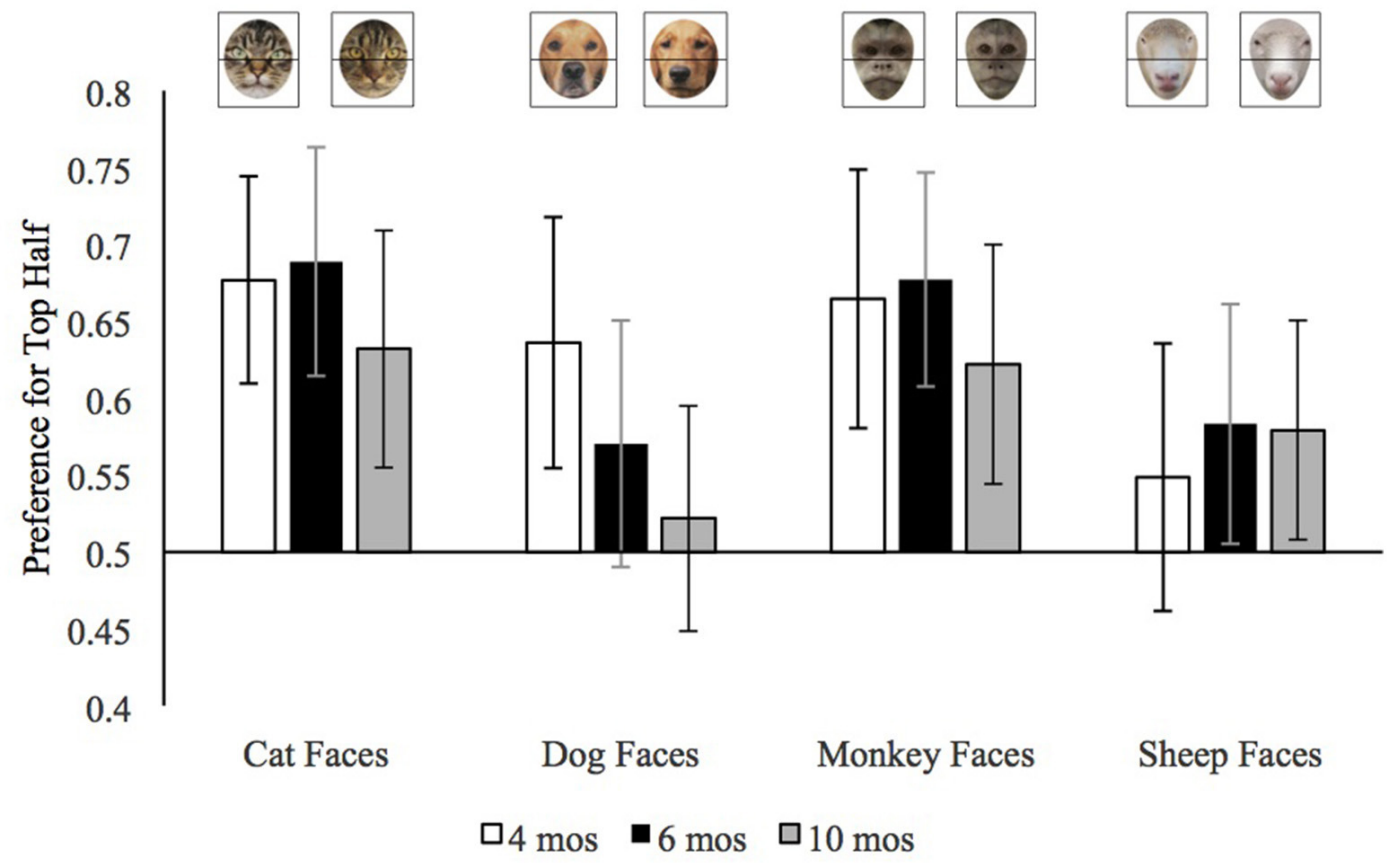
Mean preference for the top half of each face type in Experiment 2 by age. Error bars represent 95% confidence intervals.

We calculated preference for the top half of faces by dividing the looking to the top half of the face by the looking to the top and bottom half combined. We use infants' *median* top half preference across trials because the median is less influenced than the mean by extreme values. We entered each infants' median top half preference for each stimulus type (dog, cat, sheep, monkey) with pet group and age as the between-subjects variables. This analysis revealed a main effect of trial type, *F*_(3, 495)_ = 14.64, *p* < 0.001, $\eta_{\text{p}}^{2}$ = 0.08, and a trial type by age group interaction, *F*_(6, 495)_ = 2.38, *p* = 0.03, $\eta_{\text{p}}^{2}$ = 0.03. The means for this interaction are provided in Figure 3. It can be seen in this figure that, overall, there were few age differences in infants' preference for the top halves of cat, monkey, or sheep faces, but that in general older infants had a weaker preference for the top half of dog faces than did the younger infants.

To better understand the age by trial type interaction and how infants' preferences for the top halves of the different face types varied by age, we conducted separate ANOVAs on each age group. The analyses of the top half preferences by 4-month-old infants revealed only a main effect of trial type, *F*_(3, 150)_ = 7.15, *p* < 0.001, $\eta_{\text{p}}^{2}$ = 0.13. As is evident in Figure 3, 4-month-old infants (open bars) had a weaker top half preference for sheep faces than for the other faces. We confirmed this impression by conducting the mean preference scores for each of the face types, using *p* ≤ 0.008 as our cut-off for significance to control for multiple comparisons. The preference for top halves of sheep faces was significantly lower than that of cat faces, *t*_(51)_ = 4.51, *p* < 0.001, *d* = 0.63, or monkey faces, *t*_(51)_ = 3.34, *p* = 0.002, *d* = 0.46, and the difference between sheep faces and dog faces was marginal, *t*_(51)_ = 2.68, *p* = 0.01, *d* = 0.37. To provide further insight into infants' top half preferences, we compared each preference score to chance (0.50); these comparisons would confirm whether infants looked at the top half of any of the face more than expected by chance. These 4-month-old infants significantly preferred the top halves of cat faces, *t*_(51)_ = 5.23, *p* < 0.001, *d* = 0.73, dog faces, *t*_(51)_ = 3.35, *p* = 0.002, *d* = 0.46, and monkey faces, *t*_(51)_ = 3.92, *p* < 0.001, *d* = 0.55. Their preference for the top half of sheep faces did not differ from chance, *t*_(51)_ = 1.12, *p* = 0.27, *d* = 0.16. Thus, in general, at the youngest age infants preferred the top half of all the faces except the sheep faces which were both relatively unfamiliar and, as can be seen in Figure 1, dominated by the nose in the bottom half of the face.

The ANOVA on the mean preference for the 6-month-old infants also revealed only a main effect of trial type, *F*_(3, 165)_ = 8.31, *p* < 0.001, $\eta_{\text{p}}^{2}$ = 0.13, however as can be seen in Figure 3 the pattern was somewhat different. At this age, infants had a stronger preference for the top half of both cat and monkey than dog and sheep faces; cat versus dog, *t*_(56)_ = 3.97, *p* < 0.001, *d* = 0.53, monkey faces vs. dog faces, *t*_(56)_ = 3.61, *p* = 0.001, *d* = 0.48, cat vs. sheep *t*_(56)_ = 3.89, *p* < 0.001, *d* = 0.52, monkey versus sheep, *t*_(56)_ = 3.70, *p* < 0.001, *d* = 0.49. In general, 6-month-old infants seemed to have stronger preferences for the top halves of the faces that are configured with larger eyes toward the top than the faces with longer snouts and relatively large noses at the bottom.

Comparisons of the preference for the top halves to chance corroborated this conclusion. Six-month-old infants had clearly significant preferences for the top halves of cat faces, *t*_(56)_ = 5.09, *p* < 0.001, *d* = 0.67, and monkey faces, *t*_(56)_ = 5.10, *p* < 0.001, *d* = 0.68. They had non-significant preferences for the top halves of sheep faces, *t*_(56)_ = 2.14, *p* = 0.04, *d* = 0.28, and dog faces, *t*_(56)_ = 1.76, *p* = 0.08, *d* = 0.23. Bayes factor analyses confirmed that these preferences were ambiguous, at best. For the sheep faces, Bayes factor analyses with a scale r on effect size of 0.707, revealed a Scaled JZS Bayes Factor in favor of the Null of 0.85; the Scaled JZS Bayes Factor in favor of the alternative was 1.18. For the dog faces, Bayes factor analyses with a scale r on effect size of 0.707, revealed a Scaled JZS Bayes Factor in favor of the Null of 1.62; the Scaled JZS Bayes Factor in favor of the alternative was 0.62. Thus, neither the *t*-tests nor the Bayes Factor analyses provided strong support for the conclusion that 6-month-old infants preferred the top halves of dogs and sheep. In general, therefore, these 6-month-old infants preferred the top halves of faces with large eyes in the top halves, but not the top halves of faces that were dominated by long snouts.

The analysis of the top half preference by 10-month-old infants revealed significant main effects of trial type, *F*_(3, 180)_ = 4.67, *p* = 0.004, $\eta_{\text{p}}^{2}$ = 0.07, and pet group, *F*_(1, 60)_ = 4.16, *p* = 0.046, $\eta_{\text{p}}^{2}$ = 0.07. Comparisons of infants' preferences for the top halves of each type of face revealed that overall 10-month-old infants had weaker preferences for the top halves of dog faces than cat faces, *t*_(61)_ = 3.25, *p* = 0.002, *d* = 0.41, and monkey faces, *t*_(61)_ = 3.44, *p* = 0.001, *d* = 0.44; the difference between the preference for the top halves of dogs and sheep did not reach our adjusted criterion of significance, *t*_(61)_ = 2.02, *p* = 0.047, *d* = 0.26. The pet group main effect reflects the fact that across face types, 10-month-old infants with pets had stronger preferences for the top halves of faces (*M* = 0.65, SD = 0.27) than did 10-month-old infants without pets (*M* = 0.50, *SD* = 0.28). Moreover, comparison of the average top half preference to chance revealed that only infants with pets differed significantly, *t*_(39)_ = 3.52, *p* = 0.001, *d* = 0.56; the average top half preference of infants without pets was not different from chance, *t*_(21)_ = 0.14, *p* = 0.89, *d* = 0.03.

**Figure 4:**
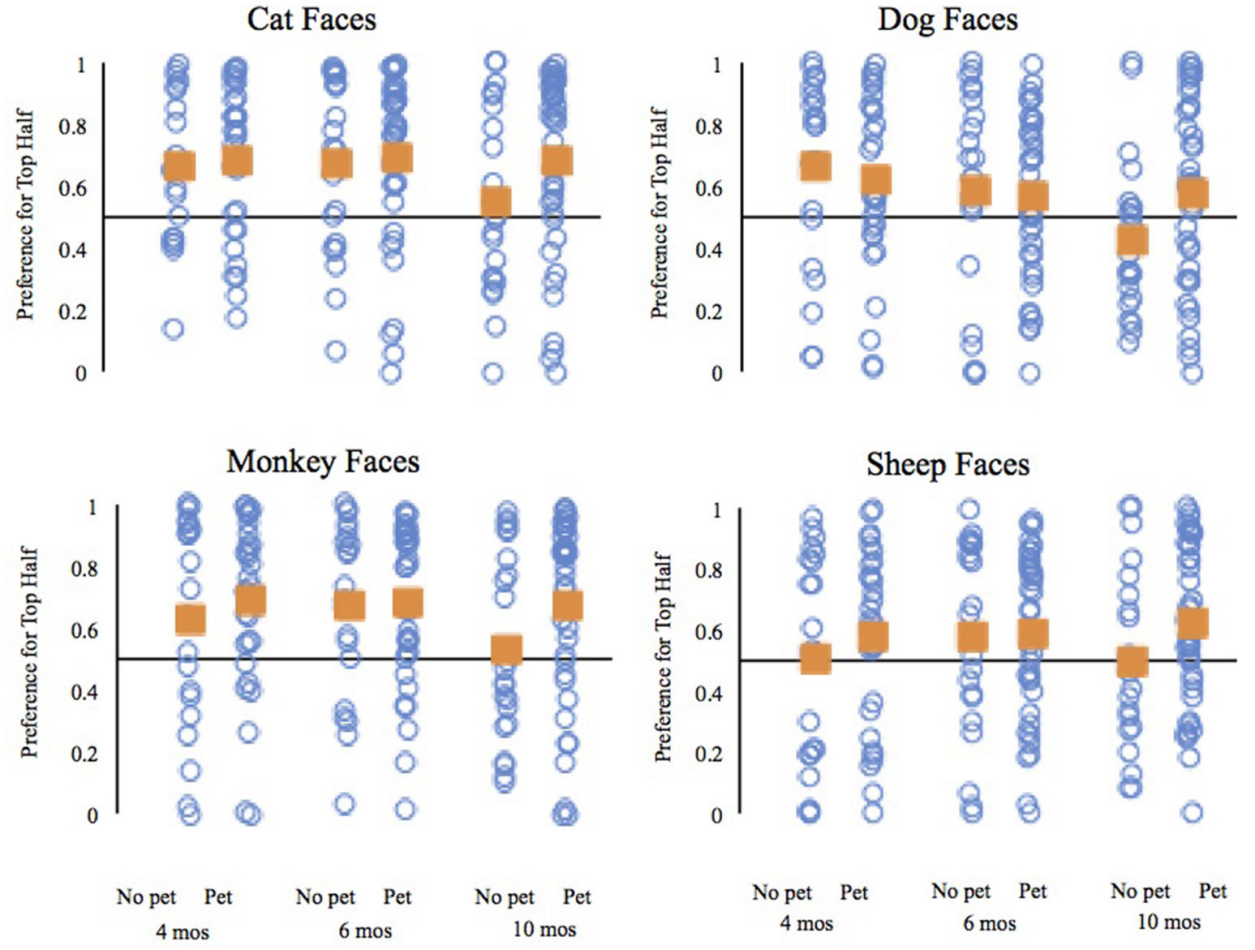
Mean preference for the top half of each face type by age and pet status. The individual blue circles represent a single infant; the squares represent the mean of each group.

Finally, to provide additional insight into the preferences of 10-month-old infants, we compared the preferences for the top halves of each type of face to chance separately for infants with and without pets (see Figure 4). The 10-month-old infants with pets significantly preferred the top halves of cat faces, *t*_(39)_ = 3.75, *p* = 0.001, *d* = 0.59, monkey faces, *t*_(39)_ = 3.55, *p* = 0.001, *d* = 0.56, and sheep faces, *t*_(39)_ = 2.85, *p* = 0.007, *d* = 0.45. Ten-month-old infants without pets did not have preferences for the top halves of any faces that significantly differed from chance; cat faces, *t*_(21)_ = 0.753, *p* = 0.46, *d* = 0.16, dog faces, *t*_(21)_ = 1.38, *p* = 0.18, *d* = 0.29, monkey faces, *t*_(21)_ = 0.54, *p* = 0.60, *d* = 0.11, and sheep faces, *t*_(21)_ = 0.03, *p* = 0.98, *d* = 0.006. Bayes Factor analyses, with an r scale of 0.707, provided modest support for the null hypothesis that the preferences were equivalent to chance for cat faces, Scaled JZS Bayes Factor of 3.47, monkey faces, Scaled JZS Bayes Factor of 3.94, and sheep faces, Scaled JZS Bayes Factor of 4.48. The Bayes Factor analysis did not provide clear support for either the null or the alternative hypothesis for the dog faces. Over time, experience with pets seems to help *maintain* an infant's interest in the top halves of these animal faces, as 10-month-old infants without pets show a reduced top half preference compared to the other age groups. All of the analyses from the 10-month-old infants lead to the same conclusion: infants with and without pets visually scanned these animal faces differently.

## General discussion

The experiments presented here provide important insight into the role of companion cats and dogs on development in infancy. Experiment 1 revealed that more than half of the infants we sampled lived with one or more pet. Thus, pets have the opportunity to have an influence on development for many infants. Experiment 2 showed that between 4 and 10 months, exposure to a pet in the home was related to how infants visually inspected images of animal faces. Although young infants with and without pets responded in the same way to animal faces, by 10 months infants with and without pets exhibited different patterns of visual inspection when looking at these images. Clearly, therefore many infants have experience with pets, and that experience seems to influence at least one aspect of their development.

These findings contribute to two separate literatures. First, they address the literature focused on the role of animals on child development (4–6, 8, 9). As described earlier, little research has examined either the prevalence of pet experience during infancy, or the effect of pet exposure on infant development. The present work addresses both gaps in the literature. In Experiment 1, we show that in our sample, ~63% of families with infants had household cats or dogs. Clearly our sample is not representative of all families, but does show that the rates of pet ownership in families with infants—at least middle-class families in the Sacramento Valley of California—are similar to those documented in other studies of pet ownership [As reported by the American Pet Products Association (56); http://www.americanpetproducts.org/press_industrytrends.asp] including in families with children (17). These data confirm that many infants have opportunities to develop in the context of experience with pets, and that this is a real difference in experience between infants. Thus, it is important to understand more about how infants' development is shaped by exposure to and experience with pets.

In addition, the data presented here confirm previous reported findings that exposure to a household cat or dog seems to induce different strategies for learning about images of dogs and cats in laboratory tasks (39–43). That is, we not only documented the prevalence of pet ownership, we also showed how infants' visual investigation of animal faces varied as a function of pet ownership. These results converge with previous findings that infants as young as 4 months visually investigate images of cats and dogs differently as a function of pet ownership (39, 42, 43), and that infants at 4 months learn about images of cats and dogs differently as a function of pet ownership (41). The results we reported here extend this previous work in several ways. First, we showed that these differences a function of pet ownership hold even when infants are shown only animal *faces*. The previous work revealed differences when infants were shown images of full bodied animals. Thus, not only does this extend previous work showing that by 4 months infants show a bias to look at the heads of whole body animal images as a function of pet experience (39, 42), it shows that previously reported results about the effect of experience on developmental changes in infants' processing and scanning of human faces (21, 27, 57, 58) may extend to the role of experience on their processing of other kinds of faces. Just as previous work suggested a tuning of human face perception between 4 and 9 months based infants' experience with face of a particular race (24, 25, 52, 59, 60), here we show a shift in the specificity of infants' investigation of animal faces as a function of their experience with dogs or cats.

Moreover, the *timing* of the effects suggests that experience with pets is not a single, unified influence, but rather that exposure to pets may have different effects at different time points. Specifically, previous work showed that pet experience influences young infants' processing of whole body images of animals (39, 42). The current results show that pet experience has an influence on infants' processing of animal faces during the same developmental time period during which infants show shifts in their perception, discrimination, and visual investigation of human faces (24, 25, 52, 54, 59–62).

Importantly, these results also show that pet experience influences not only infants' processing of familiar animals such as cats and dogs, but that daily experience with a companion animal also has an effect on infants' processing of relatively unfamiliar animals such as sheep and monkeys. Thus, the current investigation addresses the *specificity* of the effect of infants' pet experience on their face processing. Our results suggest that experience with a pet influences infants' inspection of animal faces beyond their specific pet experience, as at 10 months we observed a difference in how infants with and without pets scanned monkey faces. This effect may reflect a mechanism like that is responsible for the effect of pet experience on children's understanding of biology and living kinds (63, 64).

In summary, the results reported here add to both the literatures on the impact of animals in child development and on the effect of experience on infants' processing of visual stimuli. We showed here that pet experience is pervasive in infancy, and that this experience influences one aspect of infant development. Future research on animals and child development should not overlook the important developmental period of infancy.

## Author contributions

KH and LO both contributed to the design and implementation of the research, analysis of the results, and writing of the manuscript.

### Conflict of interest statement

The authors declare that the research was conducted in the absence of any commercial or financial relationships that could be construed as a potential conflict of interest.
