# Supplementary material for: Infants' Daily Experience With Pets and Their Scanning of Animal Faces
Source: Front Vet Sci. 2018 Jul 10;5:152. doi: 10.3389/fvets.2018.00152 (PMC6048265; doi:10.3389/fvets.2018.00152)
Supplement: Supplementary file 1 [file Table_1.DOCX]

Subject Number: __________________________ Subject Code: ___________________

__________________________ Date: __________________________

**General Infant Questionnaire**

*The following information is used to maintain accurate records of our subject population. We report demographic information in our publications as well as our funding agencies as requested. All questions are voluntary.

1. Baby’s initials (e.g. L.M.O.): __________ 2. Baby’s sex: ____ Male ____ Female

3. Is the baby Hispanic or Latino/a? ___ yes ___ no

4. What is the baby’s race (check as many as apply)?

|  | American Indian/Alaskan Native |  | Black or African American |
| --- | --- | --- | --- |
|  | Asian |  | White |
|  | Native Hawaiian or other Pacific Islander |  | Other |

If other, please specify: .

5. Birth Date: . 6. Due Date: .

7. Birth Weight: ____lbs ____oz

8. Please explain any birth complications:

.

9. Baby’s current health status (e.g. healthy, has a cold)?

.

10. Is your baby currently taking any medications? __ yes __ no

If yes, what medication? *.*

For how long? .

11. Does your child have any chronic health problems? __ yes __ no

If yes, please explain: .

12. Does your baby have any hearing problems that you are aware of? __ yes __ no

If yes, please explain: .

13. Does your baby have any vision problems that you are aware of? __ yes __ no

If yes, please explain: .

14. What is the primary language spoken at home? .

15. Is your child exposed to any other languages on a regular basis? __ yes __ no

If yes, what language(s)? .

How many hours/week or what percentage exposure for each language (approximately)?

**.**

16. Does your baby have any family members who are colorblind? __ yes __ no

If yes, who, and is the family member maternal or paternal? .

17. The person who spends the most time one-on-one with your baby is __ female __ male

18. Does someone other than a parent care for your child more than 20 hours per week? __ yes __ no

If yes, what is the gender, race, and ethnicity (to the best of your knowledge) of the person who mostly cares for your baby?

.

19. Is mother Hispanic or Latino/a? __ yes __ no

20. Is father or co-parent Hispanic or Latino/a? __ yes __ no

21. What is Mother’s race (check all that apply) 22. What is Father/co-parent’s race (check all that apply)

|  | American Indian/Alaskan Native |  | American Indian/Alaskan Native |
| --- | --- | --- | --- |
|  | Asian |  | Asian |
|  | Native Hawaiian or other Pacific Islander |  | Native Hawaiian or other Pacific Islander |
|  | Black or African American |  | Black or African American |
|  | White |  | White |
|  | Other |  | Other |

If other, please specify: . If other, please specify: .

23. What is highest degree earned by the Mother? 24. What is highest degree earned by the Father or Co-Parent?

|  | Eighth grade completion |  | Eighth grade completion |
| --- | --- | --- | --- |
|  | High School Diploma |  | High School Diploma |
|  | 2 yr degree at a college/trade school |  | 2 yr degree at a college/trade school |
|  | Some college |  | Some college |
|  | 4 yr degree from a college/university |  | 4 yr degree from a college/university |
|  | Master’s Degree |  | Master’s Degree |
|  | Doctoral Degree (Ph.D., M.D., etc) |  | Doctoral Degree (Ph.D., M.D., etc) |

25. What is the mother’s occupation? .

26. What is the father or co-parent’s occupation? .

27. Does your child have siblings? __ yes __ no *If yes, please list their* ***first*** *names, sex, & birthdates:*

| Name | Sex (M/F) | Birthdate |
| --- | --- | --- |
|  |  |  |
|  |  |  |
|  |  |  |
|  |  |  |

28. How did you hear about this research opportunity? .

29. May we contact you in the future to participate in additional studies within this lab/department?

(This may include studies for older siblings)

____ Yes ____ No
